# Adherence to healthy diet and risk of cardiovascular disease in adult survivors of childhood cancer in the St. Jude Lifetime Cohort: a cross-sectional study

**DOI:** 10.1186/s12916-023-02956-x

**Published:** 2023-07-03

**Authors:** Tuo Lan, Mei Wang, Matthew J. Ehrhardt, Shu Jiang, Jennifer Q. Lanctot, Gregory T. Armstrong, Melissa M. Hudson, Graham A. Colditz, Leslie L. Robison, Yikyung Park

**Affiliations:** 1 Division of Public Health Sciences, Department of Surgery, Washington University School of Medicine, St. Louis, MO USA; 2 Department of Oncology, St. Jude Children’s Research Hospital, Memphis, TN USA; 3 Department of Epidemiology and Cancer Control, St. Jude Children’s Research Hospital, Memphis, TN USA; 4 Alvin J. Siteman Cancer Center, Washington University School of Medicine, St. Louis, MO USA; 5 Comprehensive Cancer Center, St. Jude Children’s Research Hospital, Memphis, TN USA

**Keywords:** Dietary patterns, Cardiovascular disease, Childhood cancer survivor, Health Eating Index, DASH, Mediterranean diet

## Abstract

**Background:**

Whether diet has beneficial effects on cardiovascular disease (CVD) in childhood cancer survivors as in the general population is unknown. Therefore, we examined associations between dietary patterns and risk of CVD in adult survivors of childhood cancer.

**Methods:**

Childhood cancer survivors, 18–65 years old in the St Jude Lifetime Cohort (1882 men and 1634 women) were included in the analysis. Dietary patterns were defined by the adherence to the Healthy Eating Index (HEI)–2015, Dietary Approaches to Stop Hypertension (DASH), and alternate Mediterranean diet (aMED) based on a food frequency questionnaire at study entry. CVD cases (323 in men and 213 in women) were defined as participants with at least one grade 2 or higher CVD-related diagnosis at baseline. Multivariable logistic regression adjusted for confounders was used to estimate odds ratios (ORs) and 95% confidence intervals (CIs) of CVD.

**Results:**

Greater adherence to HEI-2015 (OR=0.88, 95% CI: 0.75–1.03, per 10 score increment), DASH (OR=0.85, 95% CI: 0.71–1.01, per 10 score increment), and aMED (OR=0.92, 95% CI: 0.84–1.00, each score increment) were, albeit trending towards significance, associated with a lower risk of CVD in women. HEI-2015 was associated with a non-significantly lower risk of CVD in men (OR_Q5 vs. Q1_=0.80, 95% CI: 0.50–1.28). These dietary patterns were also associated with a lower risk of CVD in survivors with high underlying CVD risk.

**Conclusions:**

As recommended to the general population, a diet rich in plant foods and moderate in animal foods needs to be a part of CVD management and prevention in childhood cancer survivors.

**Supplementary Information:**

The online version contains supplementary material available at 10.1186/s12916-023-02956-x.

## Background

Owing to advances in cancer treatments, survival rates of childhood cancers have significantly increased in past decades, resulting in over 480,000 childhood cancer survivors in the USA [1]. However, cancer treatments are often toxic to many organ systems, predisposing to the development of long-term chronic health conditions [2]. Adult survivors of childhood cancer have, on average, 17 chronic health conditions at age 50, which is almost twice the burden of the general population at that age [3].

In particular, childhood cancer survivors are more likely to develop severe, life-threatening, and fatal cardiovascular disease (CVD) at a younger age, making CVD the leading non-cancer morbidity and cause of mortality [4–8]. Childhood cancer survivors are eight times more likely to die from CVD than the general population [9]. The early development of CVD is, in part, due to cancer treatments such as anthracycline and chest radiation [10]. Although anthracycline and chest radiation are strong risk factors for CVD in childhood cancer survivors, other traditional CVD risk factors, for example, hypertension, dyslipidemia, and obesity are also associated with an increased risk of CVD, independent of cancer treatments [11].

Diet is an established modifiable risk factor for CVD [12]. A diet high in plant-based foods and healthy fatty acids and moderate consumption of animal-based foods, sugar, and salt, have been consistently associated with a lower risk of CVD in non-cancer individuals and high-risk individuals, such as people with diabetes, hyperlipidemia, and hypertension [13–28]. However, whether diet has similarly beneficial effects in childhood cancer survivors who are likely to have elevated underlying CVD risk is unknown. Therefore, we examined the association between dietary patterns and risk of CVD in a large number of adult survivors of childhood cancer in the St. Jude Lifetime Cohort Study (SJLIFE).

## Methods

### Study design and participants

SJLIFE was established to study the development and trajectory of health outcomes over childhood cancer survivors’ lifetimes. Details of SJLIFE have been published elsewhere [29]. Briefly, 5+ year childhood cancer survivors treated at St. Jude Children’s Research Hospital (SJCRH) in Memphis, TN were recruited. Participants of the present study are adult survivors of childhood cancer enrolled in 2007–2017. Participation involved at least one completion of clinical assessments at SJCRH, including health behaviors (e.g., smoking, alcohol intake, physical activity), psychosocial outcome surveys, and a complete medical history and physical examination, comprehensive clinical evaluations of organ function (e.g., cardiac, reproductive, neuromuscular, neurocognitive) [30, 31]. The study was approved by the St. Jude Children’s Research Hospital Institutional Review Board and all participants provided written informed consent.

Among participants ≥18 years old (*n*=4079), we excluded survivors who did not complete a dietary assessment (*n*=234); those who reported extreme energy intake (<600 or >5000 kcal/day, *n*=232); pregnant women (*n*=42); individuals with inconsistent gender records between demographics and diet questionnaires (*n*=3); and those with chronic kidney disease (*n*=26) or pancreatitis (*n*=17). As a result, 3525 participants were included in the analytic cohort.

### Dietary assessment

Dietary intake over the past 12 months was assessed using the 2005 Block Food Frequency Questionnaire (FFQ), previously validated with 24-h recalls with reasonable correlation [32, 33]. The FFQ queried 110 food items with nine categories of frequency, ranging from never to every day, as well as portion size. Pictures of foods were provided to assist with portion size estimation. Nutrient intakes were estimated using the USDA Food and Nutrient Database for Dietary Studies [34]. To comprehensively investigate the relation of a healthy dietary pattern to CVD risk, regardless of how a dietary pattern is operationally defined, three most commonly studied dietary patterns — the Healthy Eating Index (HEI)-2015, Dietary Approaches to Stop Hypertension (DASH) score, and alternate Mediterranean Diet Score (aMED) — were examined [35–38]. These dietary patterns were derived by a priori diet score or index based on a set of dietary recommendations. A summary of the dietary components and standard for scoring is presented in Additional file 1: Table S1.

#### Healthy Eating Index (HEI)-2015

HEI-2015 is a measure of diet quality in terms of conformance with the 2015–2020 Dietary Guidelines for Americans [15, 23, 35]. It includes 13 components, 9 of which assess the adequacy of consumption, including total fruit, whole fruit, total vegetables, greens and beans, whole grains, dairy, total protein foods, seafood and plant proteins, and fatty acid ratio (polyunsaturated fatty acids + monounsaturated fatty acids: saturated fatty acids). The other 4 components (refined grains, sodium, added sugars, and saturated fats) assess dietary components that should be consumed in moderation. All components, except the fatty acid ratio, were scored on a density basis (per 1000 kcal or as a percentage of energy). Intakes between the minimum and maximum standards were scored proportionately. Higher scores indicate better diet quality because the moderation components were scored such that lower intakes received higher scores. A total HEI score is a sum of each component score, which has a maximum value of 100.

#### Dietary Approaches to Stop Hypertension (DASH)

The DASH dietary pattern is rich in fruits, vegetables, and low-fat dairy and limits consumption of foods high in saturated fat, such as fatty meats and full-fat dairy products, and sugar-sweetened beverages and sweets [24, 28]. We used the method of Günther et al., which comprised 5 components that assess the adequacy of consumption, such as whole grains, fruit, vegetables, low-fat dairy, and nuts, seeds, and legumes and 3 components — meat, fats and oils, and sweets — that assess dietary components that should be consumed in moderation [36, 37]. The DASH score ranges from 0 to 80, with higher scores indicating greater adherence to DASH.

#### Alternate Mediterranean Diet (aMED)

aMED that is adapted for use in an American population has been extensively used to evaluate associations with CVD [26, 27, 38]. The score includes 9 components — vegetables (excluding potatoes), legumes, fruits, nuts, whole grains, fish, the ratio of monounsaturated fatty acids to saturated fatty acids, red and processed meats, and alcohol. One point each, except for meat and alcohol, was assigned for intake greater than the sex-specific median intake. For meat, one point was assigned for intake less than the median. For alcohol, one point was assigned for consumption between 5 and 15 g/day for women and between 10 and 25 g/day for men. The final score ranges from 0 to 9, with a higher score indicating conformity to the Mediterranean diet.

### Definition of cardiovascular disease (CVD)

All SJLIFE participants undergo comprehensive clinical assessments that provide information about symptoms, physical findings, laboratory and diagnostic testing, and clinical interventions for 190 chronic health conditions [29]. Severity of chronic conditions, including CVD, was graded based on a revised version of the NCI’s Common Terminology Criteria for Adverse Events (CTCAEv4.03) and categorized into none (grade 0), mild (grade 1), moderate (grade 2), severe/disabling (grade 3), life-threatening (grade 4) and death (grade 5) [39, 40]. We defined CVD cases as participants having at least one grade 2 or higher CVDs diagnosed at baseline. CVD included myocardial infarction, cardiomyopathy, cerebrovascular disease, vascular disease, aortic root aneurysm, stroke, cardiac dysrhythmia, conduction abnormality, right heart failure, heart valve disorder, and right ventricular systolic dysfunction.

In addition, we estimated an individual’s underlying predicted risk of CVD by age 50 using the Cardiovascular Risk Calculator (congestive heart failure, ischemic heart disease, or stroke) for childhood cancer survivors [41, 42]. We used a standard model that incorporated sex, age at cancer diagnosis, and cancer treatment history (e.g., anthracycline, chest or heart radiation) and defined a high-risk group as people with a risk score 3 or greater in one of three models, which was a commonly used cutoff used in the risk model development studies [41, 42].

### Other risk factor assessment

Participants’ sociodemographics (e.g., age, sex, race/ethnicity, education) and lifestyle (e.g., smoking, physical activity) were self-reported in questionnaires, while anthropometrics were directly measured in the SJLIFE Human Performance Laboratory. Cancer diagnosis and detailed treatment information were obtained from medical records.

### Statistical analysis

Logistic regression was used to estimate odds ratios (ORs) and 95% confidence intervals (CIs) for the association between diet pattern scores and CVD risk. HEI-2015 and DASH scores were categorized into quintiles and the aMED score was categorized into tertiles based on the distribution among the whole population. The lowest category was used as a reference. Although there was no statistically significant interaction by sex, we performed analyses in men and women separately because of sex-specific CVD risk differences. In multivariable models, we adjusted for age, race/ethnicity (non-Hispanic white, non-Hispanic black, and Hispanic or other), education (less than high school, high school graduation, training after high school, and college or post-grad), smoking (never, former, and current), alcohol (0, 0–0.49, 0.5–0.99, 1–1.99, and 2+ drinks/day), BMI (underweight, normal, overweight, and obese), physical activity (never/rarely and yes), multivitamin use (yes and no), single supplement use (yes and no), history of diabetes (yes and no), hypertension (yes and no), and high cholesterol (yes and no), and cancer treatments, including chest radiation and chemotherapy (yes and no). A linear trend across dietary pattern categories was tested by using the median value of diet score in each category in a multivariable regression model. Also, to test for nonlinearity in the associations for dietary patterns, we compared the nonparametric regression curve obtained from restricted cubic splines with a linear model using the likelihood ratio test [43]. For linear associations, we conducted analyses in which the dietary pattern score was modeled continuously. Associations of diet pattern scores with CVD risk were examined in several subgroups, such as age (<35/≥35 years), smoking (never/ever), BMI (<30/≥30 kg/m^2^), cancer treatments, without a history of hypertension, high cholesterol, or diabetes in men and women combined.

## Results

Among 1882 men [mean (SD) age: 30.6 (8.2) years] and 1634 women [mean (SD) age: 30.1 (8.0) years], there were 323 (17.2%) prevalent CVD cases in men and 213 (13.0%) in women. Time since cancer diagnosis was on average 21.7 years (SD:8.6). Mean (SD) scores for HEI-2015 (maximum 100), DASH (maximum 80), and aMED (maximum 9) were 57.8 (10.6), 36.7 (8.9), and 4.3 (2.0), respectively, in men and 62.2 (10.8), 40.2 (9.6), and 4.2 (2.0), respectively, in women. These scores were similar between the high- and low-underlying CVD risk groups. Adherence to recommended intakes for fruit, whole grains, dairy, fatty acid ratio, added sugar, meat, and sodium in HEL-2015 and DASH were low in both men and women (Additional file 1: Figure S1). Compared to men without CVD, men with CVD were older, non-White, physically inactive, and more likely to use dietary supplements, but had similar nutrient intakes from foods (Additional file 1: Table S2). On the other hand, female survivors with CVD were older and had lower nutrient intakes from foods than those without CVD.

Cancer survivors with a higher HEI-2015 score were more likely to be older, have higher educational attainment, never smoked, engage in physical activity, and use multivitamins or single dietary supplements (Table 1). The most common childhood cancer in the participants was leukemia, followed by lymphoma and central nervous system tumors. We observed some differences between men and women. Men in the highest HEI-2015 score quintile tended to be overweight and have a lower prevalence of hypertension than those in lower quintiles, while women in the highest quintile were more likely to have a healthy weight and a similar prevalence of hypertension as those in the lowest quintile. The prevalence of diabetes was highest in men in the middle quintile and women in the highest quintile. A history of receiving chest radiation was similar across quintiles in men, whereas women in the highest quintile had a higher prevalence of chest radiation therapy than those in lower quintiles. Men in the highest HEI-2015 score quintile had lower intakes of vitamin C, dietary fiber, calcium, and potassium than women in the highest quintile of the HEI-2015 score. Characteristics of participants by DASH and aMED categories were similar to those by HEI-2015 (Additional file 1: Tables S3 and S4).

**Table 1:** Characteristics of childhood cancer survivors by quintiles of Health Eating Index (HEI)-2015 in the St. Jude Lifetime Cohort Study

	Men			Women		
	Q1	Q3	Q5	Q1	Q3	Q5
Median score	(45.9)	(59.3)	(74.4)	(46.1)	(59.3)	(75.0)
*N*	472	376	276	234	322	429
Age, years (mean)	29.9	29.9	32	27.1	30.1	31.5
Race/ethnicity (%)						
White-non-Hispanic	82.8	82.7	88.8	78.2	77.6	85.8
Black-non-Hispanic	13.4	13.8	6.2	21.4	18	8.6
Others	3.8	3.5	5.1	0.4	4.4	5.6
Education^a^ (%)						
Less than high school	13.6	9.6	4.4	14.5	8.4	2.3
High school graduate	28.6	16.5	9.8	23.5	15.5	10.5
Training after high school	32.4	39.4	26.1	35.9	35.1	28.2
College or post-graduate	16.1	24.7	51.8	17.1	32.9	50.6
Other	2.8	4.0	3.3	2.1	2.8	4.2
Smoking^a^ (%)						
Never	56.1	70.0	73.9	65.8	74.5	78.6
Former	10.0	8.0	9.1	5.1	3.4	4.7
Current	22.9	12.8	8.3	21.8	17.1	7.7
Alcohol, drinks/day (%)						
0	24.8	22.9	14.5	31.2	23.6	13.8
<0.5	44.3	43.9	44.2	53.4	56.8	59.9
0.5–<1	10.8	12.0	9.1	9.0	8.7	13.5
1–<2	12.3	11.7	16.3	3.9	5.9	7.5
2+	7.8	9.6	15.9	2.6	5.0	5.4
Body mass index, kg/m^2^ (%)						
<18.5	4.9	1.3	0.7	8.1	5.0	3.3
18.5–<25	36	26.6	35.5	35.9	34.2	45.7
25–<30	23.7	39.1	37.7	21.8	21.7	23.1
30+	35.4	33.0	26.1	34.2	39.1	28.0
Physical activity, never/rarely (%)	23.9	17.0	7.6	26.5	22.4	7.9
Multivitamin use (%)	15.0	25.3	39.9	10.3	20.8	39.2
Single supplement use (%)	23.7	36.7	56.9	22.7	37.0	56.4
Hypertension (%)	9.3	9.3	6.9	6.8	13.7	7.7
High cholesterol (%)	5.3	5.1	6.9	3.9	5	6.5
Diabetes (%)	1.9	4.5	2.9	1.7	4	4.4
Cancer treatment						
Chest radiation (%)	26.3	23.1	23.2	18.4	20.8	24.2
Anthracycline (%)	56.4	58.8	57.6	63.3	55.3	55.7
Alkylating agent (%)	65.0	66.5	57.6	61.1	60.6	58.0
Platinum (%)	15.7	13.8	10.9	19.7	14.6	9.8
Cancer group (%)						
Central nervous system	17.2	16.5	10.5	13.7	14.6	8.2
Leukemia	31.6	34	35.1	36.3	39.4	38.2
Lymphoma	18.9	20.2	26.5	12.4	12.7	19.4
Wilms tumor	7.0	3.7	3.3	6.4	7.5	6.8
Other	25.4	25.5	24.6	31.2	25.8	27.5
Nutrient intake						
Total fat (% of total energy)	37.8	37.0	33.8	37.8	37.1	34.6
Vitamin C (mg/1000 kcal)	51.0	106.1	175.1	54.3	112.5	231.8
Dietary fiber (g/1000 kcal)	5.5	7.7	11.6	5.9	8.0	12.9
Calcium (g/1000 kcal)	377.3	422.8	500.7	360.1	431.5	532.8
Total sugar (g/1000 kcal)	61.0	57.7	57.7	65.1	61.6	59.0
Sodium (mg/1000 kcal)	1636.2	1671.1	1656.5	1601.1	1657.5	1680.4
Potassium (mg/1000 kcal)	1014.1	1279.8	1630.1	979.3	1260.6	1742.1

^a^Numbers may not add up to 100% due to missing

**Fig. 1:**
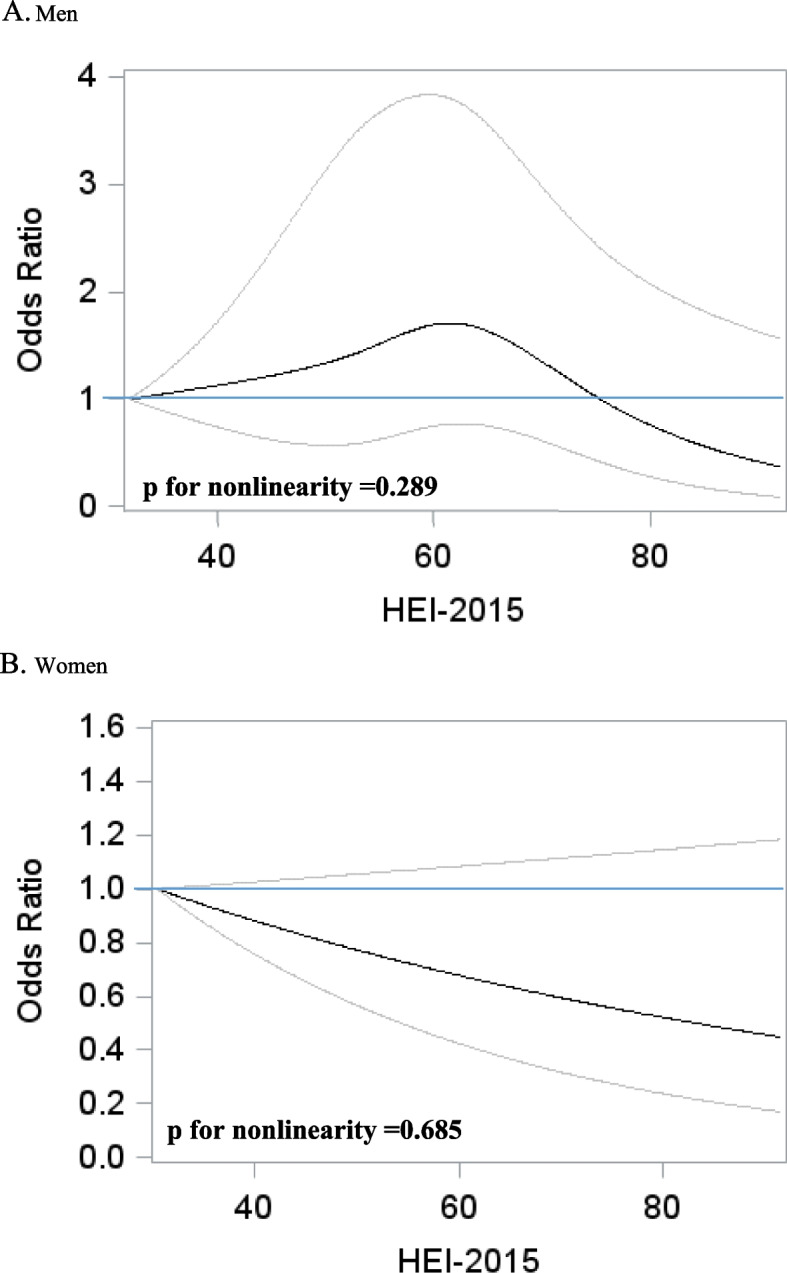
Non-parametric regression curves^1^ for associations between HEI-2015 and risk of CVD in St. Jude Lifetime Cohort Study. **A** Men. **B** Women. ^1^Adjusted for age; race (non-Hispanic white, non-Hispanic black, and Hispanic or other); education (less than high school, high school graduation, training after high school, and college or post-grad); smoking (never, former, and current); alcohol (0, 0–0.49, 0.5–0.99, 1–1.99, and 2+ drinks/day); BMI (underweight, normal, overweight, and obese); no physical activity (yes and no); multivitamin use (yes and no); single supplement use (yes and no); history of diabetes (yes and no); history of hypertension (yes and no); history of high cholesterol (yes and no); and cancer treatment receiving anthracycline (yes and no); alkylating agents (yes and no); platinum-based agents (yes and no); and chest radiation (yes and no)

In men, HEI-2015 was non-linearly associated with CVD risk (*p* for nonlinearity =0.006, Fig. 1), showing a threshold effect in high HEI-2015 scores only. Compared to men in the lowest quintile, men in the highest quintile had a lower risk of CVD (multivariable OR=0.80, 95% CI: 0.50–1.28, Table 2) that did not achieve statistical significance. DASH and aMED were not associated with CVD risk in both continuous and categorical analyses in men.

**Table 2:** Odds ratios and 95% confidence intervals of cardiovascular diseases (CVD) by categories of dietary pattern scores in the St. Jude Lifetime Cohort Study

	Continuous^a^	Quintile 1^b^	Quintile 2	Quintile 3	Quintile 4	Quintile 5	p trend
**Men**							
HEI-2015							
CVD case (*n*)	323	82	70	73	61	37	
Age-adjusted	-^c^	1.00	0.88 (0.62–1.25)	1.14 (0.80–1.62)	1.06 (0.73–1.54)	0.67 (0.44–1.03)	0.269
Multivariable^d^	-^c^	1.00	0.92 (0.64–1.33)	1.27 (0.88–1.85)	1.19 (0.80–1.76)	0.80 (0.50–1.28)	0.900
DASH							
CVD case (*n*)	323	83	68	64	57	51	
Age-adjusted	0.92 (0.81–1.06)	1.00	0.84 (0.59–1.19)	0.87 (0.61–1.25)	0.88 (0.60–1.27)	1.02 (0.69–1.51)	0.981
Multivariable^d^	1.01 (0.87–1.17)	1.00	0.91 (0.63–1.32)	1.00 (0.68–1.46)	1.09 (0.73–1.63)	1.33 (0.86–2.06)	0.185
		Tertile 1	Tertile 2	Tertile 3			
aMED							
CVD case (*n*)	323	119	107	97			
Age-adjusted	0.96 (0.91–1.02)	1.00	0.92 (0.69–1.22)	1.00 (0.74–1.35)			0.971
Multivariable^d^	0.98 (0.92–1.05)	1.00	0.97 (0.72–1.32)	1.17 (0.84–1.63)			0.391
**Women**							
HEI-2015							
CVD case (*n*)	213	36	38	41	46	52	
Age-adjusted	0.86 (0.75–0.98)	1.00	0.74 (0.45–1.22)	0.68 (0.42–1.11)	0.63 (0.39–1.02)	0.60 (0.37–0.96)	0.042
Multivariable^d^	0.88 (0.75–1.03)	1.00	0.81 (0.48–1.36)	0.72 (0.43–1.21)	0.67 (0.40–1.12)	0.68 (0.40–1.16)	0.156
DASH							
CVD case (*n*)	213	40	37	43	41	52	
Age-adjusted	0.83 (0.71–0.97)	1.00	0.81 (0.50–1.32)	0.83 (0.52–1.33)	0.63 (0.39–1.02)	0.65 (0.41–1.01)	0.038
Multivariable^d^	0.85 (0.71–1.01)	1.00	0.89 (0.54–1.49)	0.82 (0.49–1.35)	0.68 (0.40–1.13)	0.72 (0.43–1.20)	0.147
		Tertile 1	Tertile 2	Tertile 3			
aMED							
CVD case (*n*)	213	95	64	54			
Age-adjusted	0.91 (0.84–0.98)	1.00	0.68 (0.48–0.95)	0.70 (0.48–1.01)			0.036
Multivariable^d^	0.92 (0.84–1.00)	1.00	0.67 (0.47–0.97)	0.72 (0.47–1.09)			0.079

^a^Increment of 10 scores for HEI-2015 and DASH and 1 score for Mediterranean diet

^b^Median scores for HEI-2015 quintile1 to 5 were 46, 54, 59, 65, and 75 in men and women. Median scores for DASH quintile1 to 5 were 26, 33, 38, 43, and 50 in men and women. Median scores for Mediterranean diet tertile 1 to 3 were 2, 5, and 6 in men and 2, 4, and 7 in women

^c^Because the association between HEI-2015 and CVD was not linear, continuous analysis was not performed

^d^Adjusts for age; race (non-Hispanic white, non-Hispanic black, and Hispanic or other); education (less than high school, high school graduation, training after high school, and college or post-grad); smoking (never, former, and current); alcohol (0, 0–0.49, 0.5–0.99, 1–1.99, and 2+ drinks/day); BMI (underweight, normal, overweight, and obese); physical activity (never/rarely and yes); multivitamin use (yes and no); single supplement use (yes and no); history of diabetes (yes and no); history of hypertension (yes and no); history of high cholesterol (yes and no); and cancer treatment receiving anthracycline (yes and no); alkylating agents (yes and no); platinum-based agents (yes and no); and chest radiation (yes and no)

*HEI-2015* Healthy Eating Index-2015, *DASH* Dietary Approaches to Stop Hypertension, *aMED* Alternate Mediterranean diet

In women, HEI-2015, DASH, and aMED were linearly associated with a lower risk of CVD (Fig. 1 and Additional file 1: Figures S2 and S3). Each 10-score increase in HEI-2015 was associated with a 12% lowered risk of CVD (OR=0.88, 95% CI: 0.75–1.03). Each 10-score increase in DASH was also associated with a 15% lowered risk of CVD (OR=0.85, 95% CI: 0.71–1.01). aMED was also associated with an 8% (OR=0.92, 95% CI: 0.84–1.00 for 1-score increment) lower risk of CVD. Compared to the lowest category of each dietary pattern, the multivariable OR in the highest category was 0.68 (95% CI: 0.40–1.16) for HEI-2015, 0.72 (95% CI: 0.43–1.20) for DASH, and 0.72 (95% CI: 0.73–1.09) for aMED but these comparisons did not achieve statistical significance.

According to the Cardiovascular Risk Calculator for childhood cancer survivors, 1116 men (236 CVD cases) and 892 women (161 CVD cases) were classified as the high-underlying CVD risk group, and 766 men (87 CVD cases) and 751 women (52 CVD cases) were in the low-underlying CVD risk group. Compared to men in the lowest quintile of HEI-2015, men in the highest quintile had a non-significantly lower risk of CVD in both high-risk (multivariable OR=0.79, 95% CI: 0.44–1.42, *p*-trend=0.910) and low-risk (OR=0.68, 95% CI: 0.29–1.62, *p*-trend=0.705) groups (Table 3). DASH and aMED were not associated with CVD risk in both high- and low-risk groups in men. In women, greater adherence to HEI-2015 (multivariable OR=0.84, 95% CI: 0.69–1.01 per 10 score increment, *p*-trend=0.099), DASH (OR=0.79, 95% CI: 0.64–0.99 per 10 score increment, *p*-trend=0.029), and aMED (OR=0.92, 95% CI: 0.92–1.02 per one score increment, *p*-trend=0.138) was associated with a lower risk of CVD in the high-risk group but not in the low-risk group.

**Table 3:** Multivariable^a^ odds ratios and 95% confidence intervals of cardiovascular disease by categories of dietary pattern scores and underlying predicted CVD risk^b^ in the St. Jude Lifetime Cohort Study

	Continuous^c^	Quintile 1	Quintile 2	Quintile 3	Quintile 4	Quintile 5	p trend
**Men**							
HEI-2015							
High-underlying CVD risk	-^d^	1.00	1.08 (0.69–1.69)	1.27 (0.80–2.02)	1.30 (0.80–2.09)	0.79 (0.44–1.42)	0.910
Low-underlying CVD risk	-^d^	1.00	0.63 (0.30–1.28)	1.22 (0.63–2.37)	0.96 (0.44–2.08)	0.68 (0.29–1.62)	0.705
DASH							
High-underlying CVD risk	1.03 (0.85–1.23)	1.00	0.95 (0.61–1.50)	1.25 (0.79–1.98)	0.93 (0.56–1.54)	1.54 (0.90–2.61)	0.199
Low-underlying CVD risk	0.91 (0.68–1.21)	1.00	0.81 (0.40–1.63)	0.48 (0.22–1.07)	1.22 (0.59–2.52)	0.85 (0.36–1.99)	0.983
		Tertile 1	Tertile 2	Tertile 3			
aMED							
High-underlying CVD risk	1.00 (0.92–1.08)	1.00	0.94 (0.65–1.37)	1.17 (0.78–1.74)			0.484
Low-underlying CVD risk	0.93 (0.82–1.07)	1.00	1.00(0.60–1.75)	1.14 (0.60–2.18)			0.717
**Women**							
HEI-2015							
High-underlying CVD risk	0.84 (0.69–1.01)	1.00	0.79 (0.42–1.49)	0.71 (0.38–1.32)	0.54 (0.29–1.02)	0.62 (0.32–1.17)	0.099
Low-underlying CVD risk	1.00 (0.73–1.37)	1.00	1.01 (0.36–2.89)	0.58 (0.20–1.70)	1.26 (0.47–3.43)	0.96 (0.33–2.78)	0.910
DASH							
High-underlying CVD risk	0.79 (0.64–0.99)	1.00	0.72 (0.39–1.34)	0.57 (0.31–1.05)	0.62 (0.34–1.16)	0.48 (0.26–0.89)	0.029
Low-underlying CVD risk	0.91 (0.63–1.31)	1.00	0.86 (0.31–2.43)	1.26 (0.46–3.43)	0.60 (0.20–1.80)	1.26 (0.44–3.59)	0.739
		Tertile 1	Tertile 2	Tertile 3			
aMED							
High-underlying CVD risk	0.92 (0.92–1.02)	1.00	0.66 (0.43–1.03)	0.71 (0.43–1.17)			0.138
Low-underlying CVD risk	0.92 (0.77–1.11)	1.00	0.61 (0.29–1.28)	0.72 (0.31–1.70)			0.355

^a^Adjusts for age; race (non-Hispanic white, non-Hispanic black, and Hispanic or other); education (less than high school, high school graduation, training after high school, and college or post-grad); smoking (never, former, and current); alcohol (0, 0–0.49,0.5–0.99, 1–1.99, and 2+ drinks/day); BMI (underweight, normal, overweight, and obese); physical activity (never/rarely and yes); multivitamin use (yes and no); single supplement use (yes and no); history of diabetes (yes and no); history of hypertension (yes and no); history of high cholesterol (yes and no); and cancer treatment receiving anthracycline (yes and no); alkylating agents (yes and no); platinum based agents (yes and no); and chest radiation (yes and no)

^b^Individual’s underlying predicted risk of CVD by age 50 was estimated using the Cardiovascular Risk Calculator for childhood cancer survivors. A risk score 3 or greater was defined as high risk

^c^Increment of 10 scores for HEI-2015 and DASH and 1 score for Mediterranean diet

^d^Because the association between HEI-2015 and CVD was not linear, continuous analysis was not performed

*HEI-2015*, Healthy Eating Index-2015; *DASH*, Dietary Approaches to Stop Hypertension; *aMED*, Alternate Mediterranean diet

**Fig. 2:**
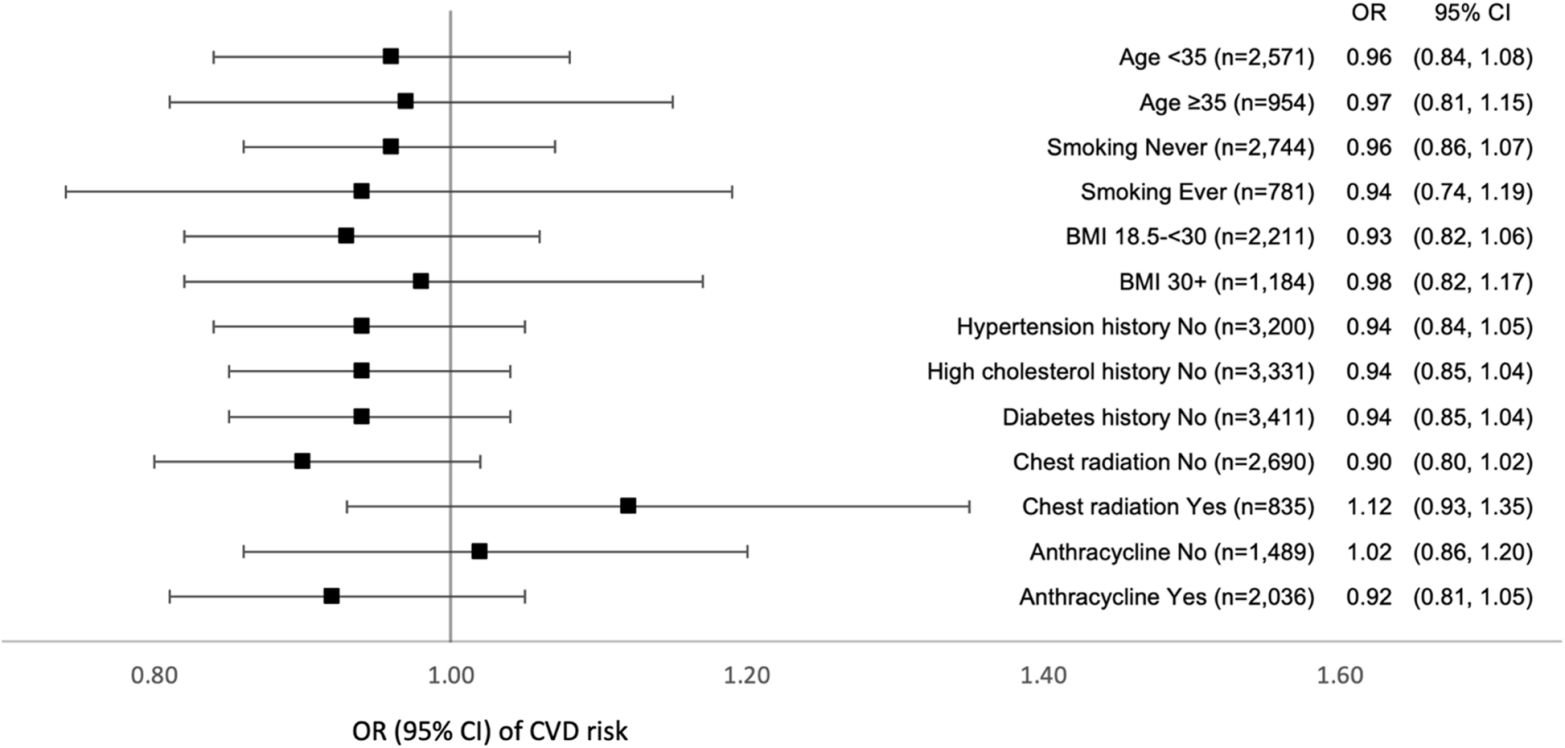
Multivariable^1^ odds ratios and 95% confidence intervals of cardiovascular disease and HEI-2015 in subgroups stratified by participants’ characteristics in the St. Jude Lifetime Cohort Study. ^1^Per 10 score increment in a multivariable model adjusted for age; race (non-Hispanic white, non-Hispanic black, and Hispanic or other); education (less than high school, high school graduation, training after high school, and college or post-grad); smoking (never, former, and current); alcohol (0, 0–0.49, 0.5–0.99, 1–1.99, and 2+ drinks/day); BMI (underweight, normal, overweight, and obese); no physical activity (yes and no); multivitamin use (yes and no); single supplement use (yes and no); history of diabetes (yes and no); history of hypertension (yes and no); history of high cholesterol (yes and no); and cancer treatment receiving anthracycline (yes and no); alkylating agents (yes and no); platinum-based agents (yes and no); and chest radiation (yes and no). The squares and horizontal lines correspond to the odds ratios and 95% confidence intervals, respectively

Subgroup analyses in men and women combined showed a consistently lower risk of CVD with higher HEI-2015 scores, albeit statistically non-significant, in all categories of age, smoking, obesity, and cancer treatments (Fig. 2). A statistically non-significant inverse association between HEI-2015 and CVD risk was also observed in people with no history of hypertension, high cholesterol, or diabetes.

## Discussion

In this large cohort of adult survivors of childhood cancer, we found that greater adherence to healthy dietary patterns, particularly HEI-2015, was associated with a lower risk of CVD, even among those survivors with high underlying CVD risk. The inverse association between healthy dietary patterns and CVD risk was more apparent in women than in men.

The preventive effect of a healthy diet on CVD risk has been well-established in non-cancer populations. Greater adherence to HEI-2015 (RR=0.83, 95% CI: 0.79–0.86 for the highest diet quality vs. lowest diet quality category), DASH (RR_Q5v.s.Q1_=0.80, 95% CI: 0.76–0.85), and aMed (RR= 0.91, 95% CI: 0.87–0.95 for a two-point increase in adherence score) was associated with a significant reduction in CVD risk in the general population [17, 22, 23, 44]. Although childhood cancer survivors in our study were much younger (mean age=30 years and median age=29 years) than the general population in studies included in meta-analyses (mean age=36–53 years and median age=52–61 years) [22, 23]. we found that conformity to healthy diet patterns in childhood cancer survivors was modestly associated with a lower risk of CVD, suggesting diet may be as impactful on CVD risk in survivors as in the general population. Likewise, a small study of adult survivors of childhood acute lymphoblastic leukemia (*n*=117, mean age=24 years at assessment) also found that the Mediterranean diet was associated with a lower risk of metabolic syndrome, a strong risk factor for CVD (age- and sex-adjusted OR=0.69, 95 % CI: 0.50–0.94 per one score increment) [45].

Previous studies reported that childhood cancer survivors had either lower, similar, or higher diet quality than their peers with no cancer [46–51]. However, all consistently found that adult survivors of childhood cancer had less than optimal diet and did not meet dietary recommendations. In our study, the average HEI-2015 score (60 out of 100) was higher than those in the general population (57 for adults 15–59 years old in the National Health and Nutrition Examination, NHANES, 2017–2018) and adult cancer survivors (55.6 for adult age >19 years in NHANES 2005–2016) [52, 53]. Nevertheless, a substantial proportion of our study participants did not consume the recommended amount of healthy foods, such as whole grains and fruits, and overconsumed unhealthy nutrients, saturated fat, and sodium (Supplementary Fig. 1). Moreover, men tended to have poorer diet quality than women, and characteristics of male cancer survivors with higher diet quality differed from those of female cancer survivors [54]. It indicates that we need to develop a sex-specific nutrition intervention and implementation strategies to prevent CVD more efficiently in long-term adult survivors of childhood cancer.

Interestingly, we found that the inverse association between a healthy diet score and risk of CVD was stronger among female cancer survivors, especially those with high underlying CVD risk, than in male cancer survivors. This finding is consistent with the results from a recent large prospective study, which reported that a poor diet quality was more strongly associated with an increased risk of CVD in women (HR=1.17, 95% CI=1.08–1.26) than in men (HR=1.07, 95% CI=0.99–1.15) [55]. This may be in part due to sex differences in disease susceptibility, cardiometabolic responses, and pathophysiology of CVD [56–58]. For example, diabetes is a stronger risk factor for CVD in women than men, and women have a lower risk of CVD than men in the general population [59]. However, previous studies on the late effects of childhood cancer suggested that female survivors of childhood cancer were more vulnerable to the adverse effects of childhood cancer treatment, including cardiotoxicity, than male survivors [3, 60]. Thus, a healthy diet may provide greater protection against CVD to female survivors with elevated CVD risk, who are more susceptible to the disease. Nonetheless, we cannot rule out the possibility of a chance finding, given that overall male survivors had poor diet than female survivors.

This study has several limitations. As the associations examined were cross-sectional, we are not able to investigate the temporal relationship between dietary patterns and CVD. Also, it is plausible that cancer survivors with prevalent CVD had changed their diet to a healthier one before the study entry, attenuating an association between dietary patterns and CVD or resulting in reverse causation. However, dietary pattern scores in survivors with CVD grade 3 or higher were similar to those with CVD grade 0 (e.g., HEI-2015 score: 57.5 in grade 3+ vs. 57.5 in grade 0 in men and 62.1 in grade 3+ vs. 61.7 in grade 0 in women), suggesting no intentional change in diet due to their disease. Nevertheless, future prospective studies are needed to replicate and confirm our findings. Another limitation inherent to the study design is insufficient power in analyses using diet score categories, the possibility of residual confounding due to inadequate adjustment of confounders, such as contextual socioeconomic factors, and measurement errors in a self-reported diet using an FFQ. To reduce the measurement errors, we excluded participants with extreme energy intake and calculated dietary pattern scores based on energy-adjusted intakes.

Despite these limitations, to our knowledge, this is the first and largest study to evaluate the relation of dietary patterns to CVD risk in a diverse group of adult survivors of childhood cancer. Remarkably few studies have examined diet and chronic diseases in long-term survivors of childhood cancer compared to a large number of studies on non-modifiable factors, such as cancer treatment, and pathophysiology. Our study suggests that diet is likely to be as effective in CVD prevention in childhood cancer survivors as in the general population. Also, our study assessed diet using a validated FFQ and featured comprehensive medical examinations for identification of CVD cases, not by self-reported diseases. Moreover, our study included childhood cancer survivors with diverse demographics, socioeconomic status, lifestyles, cancer types, and treatments, making our findings generalizable to broader childhood cancer survivor communities.

## Conclusions

Although limited, our findings support that a diet rich in fruits, vegetables, and whole grains and moderate consumption of animal-based foods, sugar, and salt may lower risk of CVD in long-term adult survivors of childhood cancer as observed in the general population. Given the high burden of CVD in childhood cancer survivors, nutrition education and dietary interventions early in survivorship care need to be a part of CVD prevention and management in this vulnerable population.

## Supplementary Information

**Additional file 1:** **Table S1.** Components and standards for scoring for Dietary Approaches to Stop Hypertension, Healthy Eating Index-2015, and Alternate Mediterranean diet. **Figure S1.** Radar plot showing the mean percentage of each component score received in men and women. **Table S2.** Characteristics of childhood cancer survivors by cardiovascular diseasestatus in the St. Jude Lifetime Cohort Study. **Table S3.** Characteristics of childhood cancer survivors by quintiles of DASH in the St. Jude Lifetime Cohort Study. **Table S4.** Characteristics of childhood cancer survivors by tertiles of Mediterranean Diet in the St. Jude Lifetime Cohort Study. **Figure S2.** Non-parametric regression curves1 for associations between DASH and risk of CVD in St. Jude Lifetime Cohort Study. **Figure S3.** Non-parametric regression curves1 for associations between Mediterranean diet and risk of CVD in St. JudeLifetime Cohort Study.

## Data Availability

Data can be obtained on request. Requests should be directed to the St. Jude LIFE (https://sjlife.stjude.org/) which has a protocol for approving data requests.

## Abbreviations

aMED: Alternate Mediterranean diet
BMI: Body mass index
CI: Confidential interval
CTCAE: Common Terminology Criteria for Adverse Events
CVD: Cardiovascular disease
DASH: Dietary Approaches to Stop Hypertension
FFQ: Food frequency questionnaire
HEI: Healthy Eating Index
OR: Odds ratio
SJCRH: St. Jude Children’s Research Hospital
SJLIFE: St. Jude Lifetime Cohort Study
