# Supplementary material for: Adherence to healthy diet and risk of cardiovascular disease in adult survivors of childhood cancer in the St. Jude Lifetime Cohort: a cross-sectional study
Source: BMC Med. 2023 Jul 3;21:242. doi: 10.1186/s12916-023-02956-x (PMC10318686; doi:10.1186/s12916-023-02956-x)
Supplement: Supplementary file 1 — Additional file 1: Table S1. Components and standards for scoring for Dietary Approaches to Stop Hypertension, Healthy Eating Index-2015, and Alternate Mediterranean diet. Figure S1. Radar plot showing the mean percentage of each component score received in men and women. Table S2. Characteristics of childhood cancer survivors by cardiovascular diseasestatus in the St. Jude Lifetime Cohort Study. Table S3. Characteristics of childhood cancer survivors by quintiles of DASH in the St. Jude Lifetime Cohort Study. Table S4. Characteristics of childhood cancer survivors by tertiles of Mediterranean Diet in the St. Jude Lifetime Cohort Study. Figure S2. Non-parametric regression curves1 for associations between DASH and risk of CVD in St. Jude Lifetime Cohort Study. Figure S3. Non-parametric regression curves1 for associations between Mediterranean diet and risk of CVD in St. JudeLifetime Cohort Study. [file 12916_2023_2956_MOESM1_ESM.pdf]

Table S1. Components and standards for scoring for Dietary Approaches to Stop Hypertension (DASH), Healthy Eating Index (HEI)-2015, and Alternate Mediterranean diet (aMED)[1]

	HEI-2015			DASH			aMED	
Component	Max Score	Standard for Maximum Score	Standard for Minimum Score	Max Score	Standard for Maximum Score	Standard for Minimum Score	Component	Criteria for 1 point
Total grains	-	-	-	5	≥6 servings/d	0 serving/d	-	-
Whole grains	10	≥1.5 oz equiv.	0 oz equiv.	5	≥50% of daily total	0% of daily total	Whole grains	> median intake
Refined grains	10	≤1.8 oz equiv.	≥4.3 oz equiv.	-	-	-	-	-
Total vegetables	5	≥1.1 cup equiv.	0 cup equiv.	10	≥4 servings/d	0 serving/d	Vegetables[2]	> median intake
Greens and beans	5	≥0.2 cup equiv.	0 cup equiv.				-	-
Total fruits	5	≥0.8 cup equiv.	0 cup equiv.	10	≥4 servings/d	0 serving/d	Fruit	> median intake
Whole fruits	5	≥0.4 cup equiv.	0 cup equiv.	-	-	-	-	-
Total dairy	10	≥1.3 cup equiv	0 cup equiv	5	≥2 servings/d	0 serving/d	-	-
Low-fat dairy	-	-	-	5	≥75% of daily total	0% of daily total	-	-
Total protein foods	5	≥2.5 oz equiv.	0 oz equiv.	-	-	-	-	-
Seafood & plant proteins	5	≥0.8 oz equiv.	0 oz equiv.	-	-	-	Fish	> median intake
Nuts-seeds-legumes	-	-	-	10	≥4 servings/wk	0 serving/wk	Nuts	> median intake
Fats-oils	-	-	-	10	≤3 servings/d	≥6 serving/d	Legumes	> median intake
Fatty acids ratio	10	(PUFAs + MUFAs) /SFAs ≥2.5	(PUFAs + MUFAs) /SFAs ≤1.2	-	-	-	MUFAs/SFAs	> median value
Meat-poultry-fish-eggs				10	≤2 servings/d	≥4 serving/d	Red and processed meats	< median intake
Saturated fats	10	≤8% of energy	≥16% of energy	-	-	-	-	-
Sweets	-	-	-	10	≤5 servings/wk	≥10 serving/wk	Alcohol	5-15g in women 5-30g in men
Added sugars	10	≤6.5% of energy	≥26% of energy	-	-	-	-	-
Sodium	10	≤1.1 gram	≥2.0 grams	-	-	-	-	-

Total score	100	80	9

1. Based on energy-adjusted intake. Intakes between the minimum and maximum standards are scored proportionately.

2. Excluding potatoes.

Figure S1. Radar plot showing the mean percentage of each component score received in men and women

A. HEI-2015

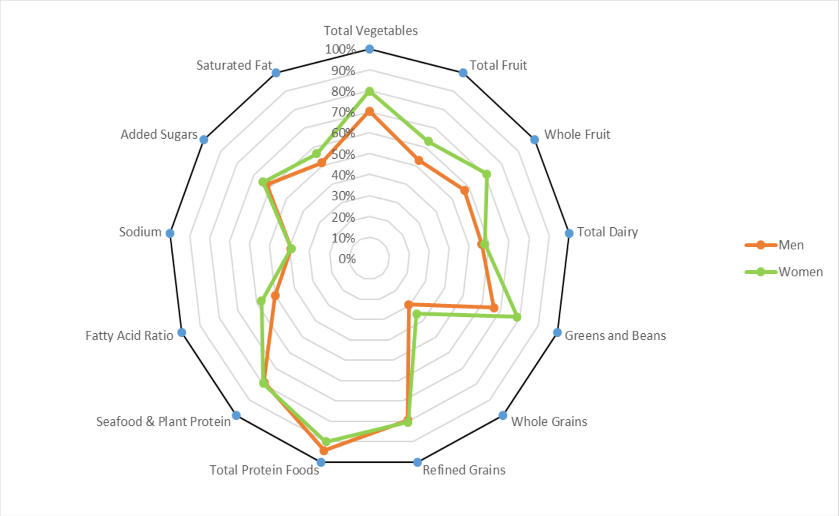

B. DASH

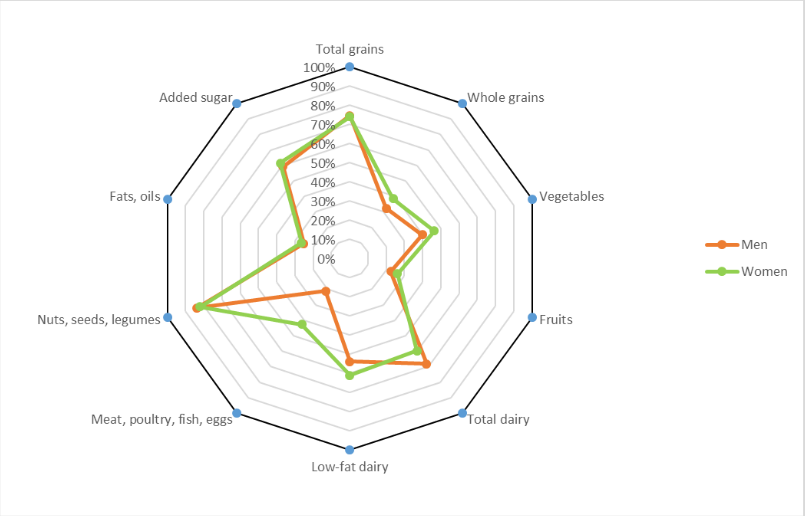

Table S2. Characteristics of childhood cancer survivors by cardiovascular disease (CVD) status in the St. Jude Lifetime Cohort Study.

	Men			Women		
	No CVD (n=1,559)	CVD (n=323)	P value	No CVD (n=1,430)	CVD (n=213)	P value
HEI-2015 (mean)	57.9	57.4	0.43	62.3	61.2	0.16
DASH (mean)	36.8	36.2	0.27	40.4	39.1	0.07
aMED (mean)	4.3	4.2	0.42	4.2	4.0	0.11
Age, years (mean)	30.1	33.0	<0.001	29.7	33.0	<0.001
Race/Ethnicity (%)						
White-non-Hispanic	84.1	80.5	0.02	80.1	82.6	0.47
Black-non-Hispanic	11.2	16.4		15.4	14.6	
Others	4.7	3.1		4.5	2.8	
Education[1] (%)						
Less than high school	9.3	12.1	0.41	7.1	8.9	0.42
High school graduate.	20.3	17.6		16.0	19.7	
Training after high school	32.6	34.1		32.9	32.4	
College or post-graduate	28.3	25.4		35.9	33.3	
Other	3.0	4.0		2.9	2.8	
Smoking (%)						
Never	66.2	62.2	0.36	73.1	70.9	0.38
Former	9.4	9.3		4.3	7.0	
Current	14.8	15.8		15.1	14.6	
Alcohol, drinks/day (%)						
0	20.8	23.8	0.57	20.6	23.0	0.86
<0.5	43.9	44.0		56.2	56.3	
0.5 - <1	11.1	10.8		11.6	9.9	
1 - <2	12.8	9.9		6.6	5.6	
2+	11.4	11.5		5.0	5.2	
Body mass index, kg/m^2 (%)						
<18.5	2.1	4.6	0.06	4.8	6.1	0.69
18.5 - <25	31.2	31.6		38.0	37.6	
25 - <30	32.9	30.3		24.0	21.1	
30+	33.7	33.4		33.2	35.2	
No physical activities (%)	14.4	24.1	<0.001	18.0	26.3	0.004
Multivitamin use (%)	23.0	30.7	0.003	26.0	27.7	0.60
Single supplement use (%)	34.9	42.1	0.01	39.7	49.3	0.01
Hypertension (%)	7.5	15.8	<0.001	8.7	15.0	0.004
High cholesterol (%)	4.9	11.8	<0.001	4.2	9.4	0.001
Diabetes (%)	3.0	3.7	0.47	3.6	1.9	0.19
Cancer treatment						
Chest radiation (%)	22.1	35.6	<0.001	21.0	35.7	<0.001
Alkylating agent (%)	62.9	71.5	0.003	59.0	68.5	0.008
Anthracycline (%)	57.3	61.0	0.27	57.0	61.0	0.27

Platinum (%)	12.8	20.1	<0.001	12.7	18.8	0.01
Cancer group (%)						
Central nervous system tumors	15.7	13.9	<0.001	11.5	13.1	0.002
Leukemia	35.7	25.4		38.3	25.4	
Lymphoma	18.7	27.6		15.5	23.5	
Other	25.7	27.6		27.2	31.0	
Wilms tumor	4.3	5.6		7.6	7.0	
Nutrient intake						
Total fat (%of total energy)	36.6	37.1	0.09	36.3	37.1	0.06
Vitamin C (mg/1,000 kcal)	95.4	106.2	0.25	137.7	144.0	0.72
Dietary fiber (g/1,000 kcal)	7.7	7.5	0.28	9.3	8.8	0.06
Calcium (g/1,000 kcal)	422.6	420.9	0.83	452.0	429.5	0.05
Total sugar (g/1,000 kcal)	58.7	58.7	0.99	61.0	59.7	0.40
Sodium (mg/1,000 kcal)	1652.9	1668.0	0.33	1661.6	1644.4	0.37
Potassium (mg/1,000 kcal)	1254.0	1252.0	0.91	1377.3	1315.6	0.02

1. Numbers may not add up to 100% due to missing.

Table S3. Characteristics of childhood cancer survivors by quintiles of DASH in the St. Jude Lifetime Cohort Study

	Men			Women		
	Q1	Q3	Q5	Q1	Q3	Q5
Median	26	38	50	27	38	50
N	449	393	264	256	313	443
Age, years(mean)	30.7	30.3	31.6	29.1	29.4	31.0
Race/Ethnicity (%)						
White-non-Hispanic	80.2	84.7	88.3	70.3	79.6	86.5
Black-non-Hispanic	16.5	10.9	6.1	28.1	15.7	7.2
Others	3.3	4.3	5.7	1.6	4.8	6.3
Education[1] (%)						
Less than high school	12.9	8.7	6.8	17.2	5.4	2.0
High school graduate.	26.9	17.6	10.6	22.3	14.4	9.7
Training after high school	34.7	33.1	26.1	35.9	39.0	29.8
College or post-graduate	15.6	31.6	48.9	16.8	33.2	49.7
Other	2.4	3.1	2.3	1.6	2.9	3.8
Smoking[1] (%)						
Never	52.8	71.0	80.7	61.3	75.4	77.0
Former	10.5	10.9	4.5	4.7	3.2	5.0
Current	24.7	10.2	7.2	27.0	15.7	8.8
Alcohol, drinks/day (%)						
0	22.5	21.4	21.2	28.5	22.7	14.0
<0.5	44.5	43.8	43.6	53.9	53.4	58.0
0.5 - <1	9.4	10.4	9.8	7.8	12.1	15.1
1 - <2	13.1	15.0	14.0	5.1	6.7	7.9
2+	10.5	9.4	11.4	4.7	5.1	5.0
Body mass index, kg/m^2 (%)						
<18.5	4.0	2.0	1.1	9.4	5.8	4.1
18.5 - <25	38.1	29.0	34.8	35.2	38.0	43.6
25 - <30	25.2	36.6	36.4	21.5	23.6	23.5
30+	32.7	32.3	27.7	34.0	32.6	28.9

No physical activities (%)	23.8	12.7	8.0	32.0	18.2	10.8
Multivitamin use (%)	15.6	26.2	37.5	10.9	24.9	39.3
Single supplement use (%)	22.7	38.7	56.1	21.1	40.6	55.1
Hypertension (%)	10.7	8.9	9.8	7.8	11.8	7.7
High cholesterol (%)	5.8	7.4	8.3	4.3	4.5	6.8
Diabetes (%)	2.4	4.6	4.2	2.0	2.9	4.5
Cancer treatment						
Chest radiation (%)	26.5	24.2	24.2	22.7	29.1	23.3
Alkylating agent (%)	61.5	65.1	62.1	64.8	62.9	59.4
Anthracycline (%)	57.7	58.8	57.2	64.1	56.9	55.3
Platinum (%)	14.3	14.2	10.6	15.6	16.9	11.7
Cancer group (%)						
Central nervous system tumors	13.8	17.6	13.3	8.6	16.0	8.6
Leukemia	31.6	36.1	37.9	35.9	32.3	38.1
Lymphoma	18.9	19.8	23.9	19.9	16.3	18.1
Wilms tumor	7.6	4.3	1.9	6.3	6.4	8.4
Other	28.1	22.1	23.1	29.3	29.1	26.9
Nutrient intake						
Total fat (%of total energy)	37.1	37.3	33.4	36.8	37.5	33.6
Vitamin C (mg/1,000 kcal)	61.3	90.7	164.9	70.8	115.5	219.6
Dietary fiber (g/1,000 kcal)	5.4	7.7	11.6	5.9	8.3	12.8
Calcium (g/1,000 kcal)	355.0	418.0	537.9	322.9	433.2	553.7
Total sugar (g/1,000 kcal)	67.2	55.0	57.8	72.7	58.0	60.9
Sodium (mg/1,000 kcal)	1558.1	1688.6	1716.9	1533.5	1662.3	1699.2
Potassium (mg/1,000 kcal)	1022.7	1258.9	1636.7	1007.9	1283.6	1741.2

1. Numbers may not add up to 100% due to missing.

Table S4. Characteristics of childhood cancer survivors by tertiles of Mediterranean Diet in the St. Jude Lifetime Cohort Study

	Men			Women		
	T1	T2	T3	T1	T2	T3
Median	2	5	6	2	4	7
N	689	655	538	643	567	433
Age, years(mean)	30.0	30.5	31.4	28.7	30.3	31.9
Race/Ethnicity (%)						
White-non-Hispanic	83.2	83.5	83.8	77.6	81.7	82.9
Black-non-Hispanic	12.6	12.7	10.6	20.2	13.1	10.9
Others	4.2	3.8	5.6	2.2	5.3	6.2
Education[1] (%)						
Less than high school	13.4	9.9	5.0	11.5	5.5	3.5
High school graduate.	27.1	19.8	10.6	22.4	16.4	7.9
Training after high school	34.1	33.4	30.5	35.8	30.9	31.2
College or post-graduate	15.4	26.1	45.7	22.4	39.3	50.1
Other	2.5	4.1	2.8	2.5	2.3	4.2
Smoking[1] (%)						
Never	58.6	65.8	74.0	69.2	74.3	76.4
Former	10.0	9.8	8.0	4.5	4.6	5.1
Current	20.9	14.0	8.4	20.2	13.4	9.5
Alcohol, drinks/day (%)						
0	26.9	20.5	15.4	28.0	20.6	10.9
<0.5	47.3	43.5	40.1	56.9	55.0	56.6
0.5 - <1	6.1	13.0	15.1	5.0	11.3	21.0
1 - <2	8.0	13.1	16.7	5.3	7.2	7.4
2+	11.8	9.9	12.6	4.8	5.8	4.2
Body mass index, kg/m^2 (%)						
<18.5	3.3	2.6	1.5	5.3	5.1	4.4
18.5 - <25	32.7	28.7	32.7	35.3	36.5	43.6
25 - <30	27.6	35.1	35.5	22.4	24.3	24.5
30+	36.4	33.6	30.3	37.0	34.0	27.5

No physical activities (%)	21.8	14.5	10.8	26.6	17.5	10.2
Multivitamin use (%)	17.1	24.4	33.3	18.0	26.1	38.6
Single supplement use (%)	26.1	36.0	49.1	29.2	42.2	56.6
Hypertension (%)	8.7	9.8	8.2	10.3	9.9	8.1
High cholesterol (%)	5.4	7.0	5.8	4.0	3.9	7.4
Diabetes (%)	2.5	4.0	2.8	2.8	3.7	3.9
Cancer treatment						
Chest radiation (%)	25.4	24.0	23.6	19.4	24.2	26.3
Alkylating agent (%)	64.9	64.0	64.1	59.4	63.0	57.7
Anthracycline (%)	57.3	57.3	59.7	61.3	54.9	55.4
Platinum (%)	15.5	13.6	12.6	14.5	14.3	10.9
Cancer group (%)						
Central nervous system tumors	16.1	15.7	13.9	12.8	11.6	10.2
Leukemia	33.4	35.3	33.1	38.7	36.7	33.3
Lymphoma	19.6	18.2	23.4	14.5	14.8	21.9
Wilms tumor	5.7	4.4	3.2	7.6	6.9	8.1
Other	25.3	26.4	26.4	26.4	30.0	26.6
Nutrient intake						
Total fat (% of total energy)	37.3	36.6	35.9	37.1	36.4	35.4
Vitamin C (mg/1,000 kcal)	67.1	95.6	137.8	89.4	143.2	205.2
Dietary fiber (g/1,000 kcal)	5.9	7.7	9.9	6.9	9.5	12.4
Calcium (g/1,000 kcal)	405.3	424.6	441.3	416.4	456.9	487.3
Total sugar (g/1,000 kcal)	61.4	58.6	55.2	64.3	59.8	57.0
Sodium (mg/1,000 kcal)	1617.3	1657.3	1702.2	1612.9	1665.2	1720.6
Potassium (mg/1,000 kcal)	1097.3	1257.8	1448.8	1150.4	1400.0	1654.3

1. Numbers may not add up to 100% due to missing.

Figure S2. Non-parametric regression curves[1] for associations between DASH and risk of CVD in St. Jude Lifetime Cohort Study

A. Men

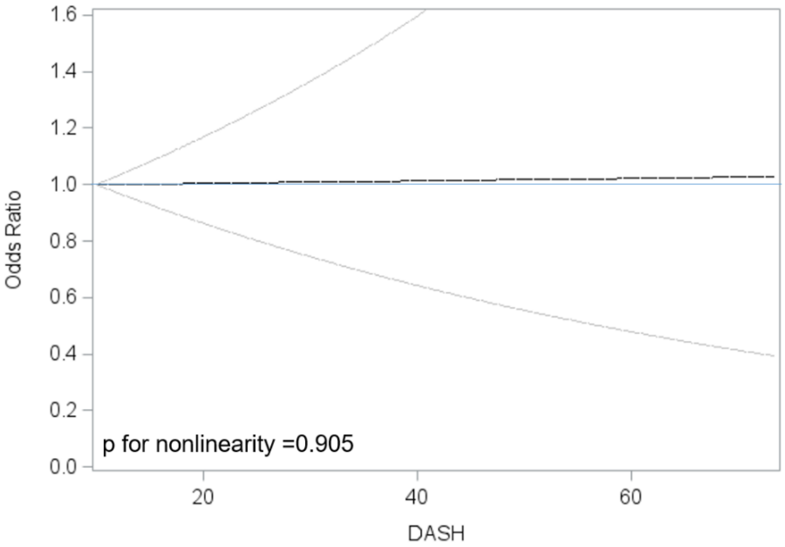

B. Women

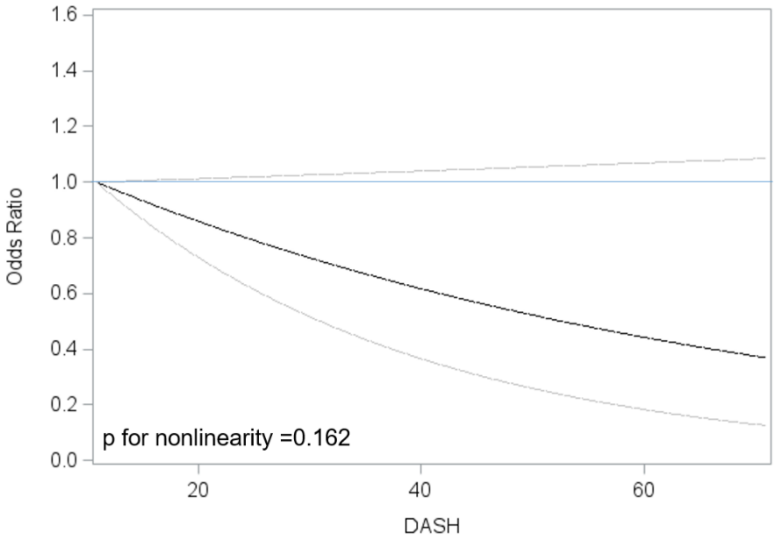

1. Adjusted for age; race (non-Hispanic white, non-Hispanic black, and Hispanic or other); education (less than high school, high school graduation, training after high school, and college or post-grad); smoking (never, former, and current); alcohol (0, 0-0.49,0.5-0.99.1-1.99, and 2+ drinks/day); BMI (underweight, normal, overweight, and obese); no physical activity (yes and no); multivitamin use (yes and no); single supplement use (yes and no); history of diabetes (yes and no); history of hypertension (yes and no); history of high cholesterol (yes and no); and cancer treatment receiving anthracycline (yes and no); alkylating agents (yes and no); platinum based agents (yes and no); and chest radiation (yes and no).

Figure S3. Non-parametric regression curves[1] for associations between Mediterranean diet and risk of CVD in St. Jude Lifetime Cohort Study

A. Men

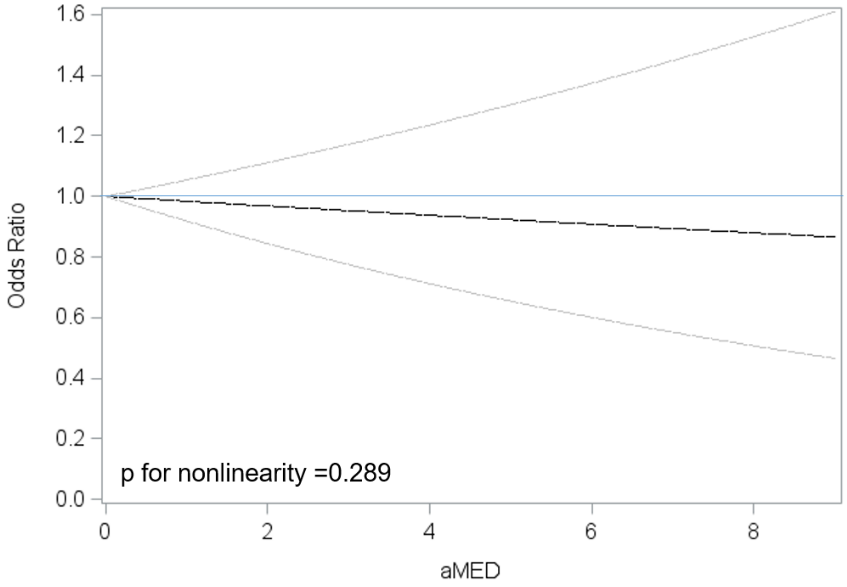

B. Women

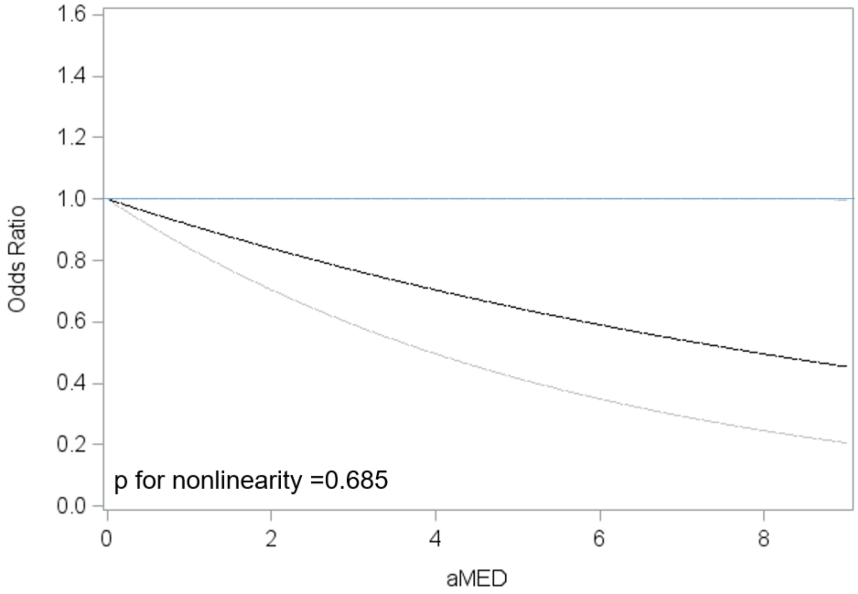

1. Adjusted for age; race (non-Hispanic white, non-Hispanic black, and Hispanic or other); education (less than high school, high school graduation, training after high school, and college or post-grad); smoking (never, former, and current); alcohol (0, 0-0.49,0.5-0.99.1-1.99, and 2+ drinks/day); BMI (underweight, normal, overweight, and obese); no physical activity (yes and no); multivitamin use (yes and no); single supplement use (yes and no); history of diabetes (yes and no); history of hypertension (yes and no); history of high cholesterol (yes and no); and cancer treatment receiving anthracycline (yes and no); alkylating agents (yes and no); platinum based agents (yes and no); and chest radiation (yes and no)
